# Survival persistence of the 3 common *Salmonella enterica* serotypes isolated from broilers' in different matrices

**DOI:** 10.1016/j.psj.2023.102788

**Published:** 2023-05-24

**Authors:** Marwa Fawzy ElMetwaly Ahmed, Bussarakam Chuppava, Jan Berend Lingens, Julia Hankel, Amr Abd El-Wahab, Pia Münster, Ali Antakli, Dimitri Radko, Christian Visscher

**Affiliations:** ⁎ Hygiene and Zoonoses Department, Faculty of Veterinary Medicine, Mansoura University, 35516 Mansoura, Egypt; † Institute for Animal Nutrition, University of Veterinary Medicine Hanover, Foundation, 30173 Hannover, Germany; ‡ Department of Nutrition and Nutritional Deficiency Diseases, Faculty of Veterinary Medicine, Mansoura University, 35516 Mansoura, Egypt; § Elanco Deutschland GmbH, 61352 Bad Homburg, Germany

**Keywords:** broiler, *Salmonella*, persistence, feeding line, in vitro

## Abstract

Broiler meat is the predominant source of *Salmonella* as a foodborne pathogen. Several control strategies have focused on the reduction of *Salmonella* spp. levels at different production stages. However, the persistence of *Salmonella* between consecutive flocks is still of great concern. This study was designed to understand the cause of reinfection in broiler flocks due to survival of *Salmonella* in feeding lines of related matrices. *Salmonella* (***S.***) Enteritidis, *S.* Infantis, and *S.* Typhimurium isolated from broiler farms in North-West Germany were used. Four types of matrices (phosphate buffer saline (**PBS**), dietary plant fat, fat with feed mixture, and feed) were applied to evaluate *Salmonella* survival (with the initial dose about 8.0 log_10_ CFU/mL) during a simulation of 4 production cycles. To evaluate the growth and survival status of *Salmonella* ISO 6579-1:2017 were performed (quantitatively by plate count method (**PCM**) and most probable number method (**MPN**)) and qualitatively) at 5 defined time points (−7, 0, 4, 7, and 35 d). In all matrices and for the 3 serovars, the *Salmonella* count decreased at the end of the fourth cycle in comparison to the beginning of the experimental infection, and was still cultivated except for fat matrix. The PBS matrices showed the highest survival level of *Salmonella* and did not decline drastically by the end of the fourth cycle (5.93 ± 0.00, 5.87 ± 0.02, 5.73 ± 0.05 log_10_ CFU/mL, respectively). However, the fat matrices showed the lowest survival level for the 3 isolates at d 35 since the first cycle (0 log_10_ CFU/mL using PCM). Regarding the fat-feed mixture, and feed matrices, there was a fluctuation in the survival rate of *Salmonella* (all serovars) within each cycle. For the qualitative method, the 3 serovars persisted in all matrices until the end of the fourth cycle except for fat matrices. The present study highlights the ability of *Salmonella* to survive for a long time in different temperatures and matrices despite efficient cleaning and disinfection processes in the feeding lines, which may influence reinfection with *Salmonella* in poultry houses.

## INTRODUCTION

Consumption of contaminated chicken meat was reported as a major source of several foodborne diseases, among which *Salmonella* (***S*.**) spp. was one of the main etiological agents (EFSA and ECDC, 2019). The most commonly reported serovars causing human salmonellosis in the European Union (**EU**) in 2018 were *S.* Enteritidis, *S.* Typhimurium, and *S.* Infantis (EFSA and ECDC, 2019).

Furthermore, the main risks of *Salmonella* horizontal transmission include residual presence of pathogens from the preceding flocks or by other environmental sources such as: contaminated feed or drinking water, pests, wild birds, insects, litter and even personnel, equipment and vehicles (Pulido-Landínez, 2019). Thus, inadequate cleaning and disinfection of broiler houses as well as stable equipment can promote the risk of infection with *Salmonella* and lead to contamination within the flock or the following flock (Rose et al., 2000). Due to the persistence of *Salmonella* in the contaminated stables and equipment, reinfection can occur (Rose et al., 2000).

It is well known that *Salmonella* is able to present in the feed to multiply in warm, moist surroundings (feed mill or on the farm), however, dietary fat tend to protect *Salmonella* from environmental or physiological stress, and cannot be easily eliminated (Wason et al., 2021). Consequently, reducing fat accumulation in the feeding lines is important to decrease the chances of *Salmonella* survival and spread (Jones, 2011).

Prevention of *Salmonella* contamination in the broiler production chain is very important, especially at preharvest level, as it could be beneficial in decreasing the prevalence of *Salmonella* infection in general. The risk factors associated with *Salmonella* infection at preharvest, processing and postprocessing levels have been well characterized, but their mechanisms of persistence and/or spread in poultry systems are largely unknown. The aim of this preliminary was to run a model under laboratory conditions to gain an idea of the ability of *Salmonella* serovars to survive in broiler feed residues present in feeding lines in practice.

## MATERIALS AND METHODS

### Bacterial Strains and Inoculum Preparation

*Salmonella enterica* subsp. *enterica* serovar Enteritidis (*S*. Enteritidis), *S. enterica* subsp. *enterica* serovar Infantis (*S*. Infantis), and *S. enterica* subsp. *enterica* serovar Typhimurium (*S*. Typhimurium) obtained from AniCon Labor GmbH (Höltinghausen, Germany) were used in this study. The 3 isolates had been previously isolated from poultry farms associated with Elanco Deutschland GmbH (Bad Homburg, Germany) in terms of diagnostics during routine biosecurity measure checks on the farms, the bacterial identification was performed until the serotype level. To prepare the bacterial suspension, the test strain was streaked on Columbia sheep blood agar (Thermo Scientific, Thermo Fisher Scientific GmbH, Wesel, Germany) at 37°C for 24 h. After incubation to obtain bacterial suspension at a concentration of 8.0 log_10_ CFU/mL, colony material was suspended in NaCl 0.9% (B. Braun Melsungen AG, Melsungen, Germany) until a McFarland grade of 0.5 was reached (McFarland densitometer DEN-1B, BioSan SIA, Riga, Latvia). Simultaneously, the bacterial concentration of the inoculated dose was verified by direct plating of appropriate dilutions of the suspension.

### Matrix Samples

**Figure 1:**
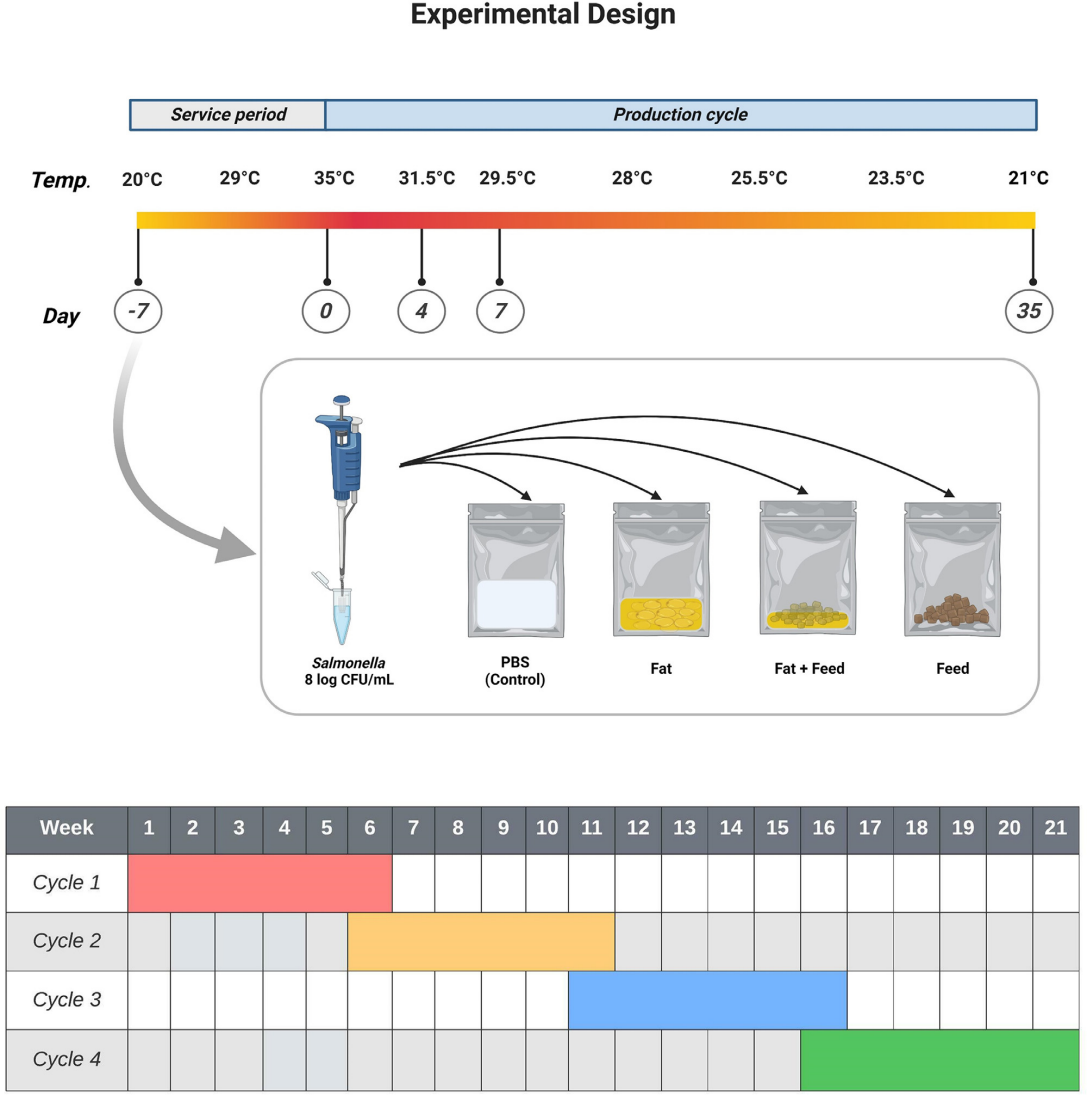
Overview concerning the temperature and sample plan for *Salmonella* diagnostic. *Salmonella enterica* subsp. *enterica* (with the initial dose ~ 8.0 log10 CFU/mL) were added to the 4 matrices; phosphate buffer saline (PBS), dietary plant fat, fat with feed mixture (1:10), and feed. To evaluate the growth and survival status of *Salmonella*, plate count method (PCM) and most probable number method (MPN) were performed at 5 defined time points (−7, 0, 4, 7, and 35 d) for 4 consecutive production cycles. Abbreviation: CFU, colony forming unit. Figure was created with BioRender.com (30 January 2022).

Four types of matrices were used for detection of *Salmonella* survival during 4 simulated production cycles. In total, 20 Whirl-Pak bags (Whirl-Pak, Nasco International Inc., Fort Atkinson, WI) (5 bags for each matrix) were used for each strain in each production cycle (a total of 240 bags were prepared at the beginning of the experiment). The PBS served as control group; dietary plant fat used in the poultry diet was obtained from MEGA Tierernaehrung GmbH & Co.KG, fat with feed mixture was obtained by mixing fat and feed in a ratio of 1:10 (2.25 g of fat and 20.25 g of feed; Figure 1), while the last group, feed contained starter poultry and was obtained from the Farm for Education and Research in Ruthe, University of Veterinary Medicine Hannover, Foundation, Hannover, Germany. The commercial starter diet was based on wheat grain, yellow corn, soybean meal, and rapeseed meal obtained from a local feed company (MEGA Tierernährung GmbH & Co. KG, Visbek-Rechterfeld, Germany). Each sample, 22.5 mL or g, was weighed into Whirl-Pak bags. In order to rule out *Salmonella* contamination, before the start of the experiment, from the feed and fat matrices used, qualitative analyses in accordance with ISO 6579-1:2017 had been carried.

### Experimental Set-Up

All the bags of the 4 matrices (*n* = 240, 20/production cycle/strain) for the 4 production cycles were prepared at the beginning of the experiment. The matrices were artificially contaminated with *Salmonella* at the beginning of the service period (7 d before the start of the first production cycle (d −7)). All inoculated matrices were exposed to the different temperatures of the 4 simulated production cycles during the entire experimental period (147 d, d −7 until d 35/cycle). The contaminated matrices were prepared by inoculating them separately with 2.5 mL of a broth containing *Salmonella* (*S.* Enteritidis, *S.* Infantis, and *S.* Typhimurium) approximately 8.0 log_10_ CFU/mL in 22.5 mL or g of each sample (1:10) (PBS, fat, fat-feed mixture, and feed) which was mixed thoroughly by kneading and shaking in a sealed Whirl-Pack bag. The inoculated matrices were kept in the incubator equivalent to the temperature in the broiler stable (simulation of the service periods (from 20°C increased to 35°C) and production cycles (from 35°C decreased to 21°C, Figure 1). The contamination experiment was conducted with the 3 *Salmonella* strains according to the same investigation scheme (Figure 1). To determine the growth and survival of *Salmonella* in the matrices (PBS, fat, fat with feed mixture, and feed), the samples were used in a 7-day experiment for the service period and 35-day experiment for each production cycle. At defined time points (at −7, 0, 4, 7 and 35 d as shown in Figure 1); the 3 *Salmonella* strains were examined microbiologically by quantitative and qualitative methods to evaluate the growth and survival status of the *Salmonella*.

### *Salmonella* Diagnostic Method

*Salmonella* investigations were carried out for all samples quantitatively and qualitatively in accordance with ISO 6579-1:2017. The following describes the methods for the qualitative and quantitative evaluation: At each sampling time for each matrix, 1 Whirl-Pak bag was used for quantitative and qualitative determination of *Salmonella*. Each sample at each time point was added with peptone water (**PW**; Thermo Scientific, Thermo Fisher Scientific GmbH) in a ratio of 1:10 and mixed using Bagmixer (BagMixer400, Interscience SARL, Saint-Nom-la-Bretèche, France) for 90 s at speed level 3. This mixture was the first dilution step after of the suspension. Tween 80 was added to the matrix according to the amount of fat present in each matrix at a ratio of 1 g/L for each 10% fat in the matrices in accordance with Norm DIN EN ISO 6887-1 (July 2017).

### Qualitative Detection of *Salmonella*

For the qualitative detection of *Salmonella*, after incubating the PW from the first dilution step for 24 h at 37°C, each sample was streaked onto selective culture media xylose lysine deoxycholate agar (**XLD**, Thermo Scientific, Thermo Fisher Scientific GmbH) and stored for 24 h in the incubator at 37°C.

### Quantitative Detection of *Salmonella*

#### Plate Counting Method

One milliliter of the first dilution step was transferred to a deep well block (96 well MegaBlock. RTM. 2.2 mL, Sarstedt AG & Co., Nümbrecht) and thereafter, 10-fold serial dilution (100 µL sample in 900 µL peptone water) until dilution step 8. In the second step, aliquots (100 µL) from sample suspensions of the 10-fold dilution series were plated in duplicate onto XLD agar (Thermo Fisher Scientific GmbH). After incubating the media for 24 h at 37°C, the characteristic black *Salmonella* colonies were counted, and the results were expressed in log_10_ CFU/mL or g.

#### Most Probable Number Method

In addition to the qualitative and quantitative analysis with the plating method, the most probable number (**MPN**) method was performed to detect *Salmonella*. The latter was performed as described by Pavic et al. (2010), where the bacterial count of the sample material was determined by a serial dilution and a nonselective enrichment of the individual dilution stages in PW. In principle, qualitative proof of the dilution steps was carried out in triplicate and subsequently the number of bacteria was calculated by a software program.

In detail, the mixture of the first dilution step after homogenization of the suspension was transferred to a deep well block (96 well MegaBlock. RTM. 2.2 mL). This was followed by a logarithmic dilution series of the sample with peptone water in a deep well block (96 well MegaBlock. RTM. 2.2 mL) in which an 8-stage dilution series (100 μL sample: 900 μL peptone water) was prepared. From the finished dilution series, 100 μL of each dilution were transferred in triplicate to a microtiter plate (96 well microtest plate, Sarstedt AG & Co, Numbrecht). After incubation for 24 h at 37°C, the total volume of each well was transferred to the corresponding well of another deep well block filled with 500 μL Modified Semisolid Rappaport Vassiliadis (**MSRV**) Medium supplemented with MSRV selective supplement SR0161E (Thermo Scientific, Thermo Fisher Scientific GmbH) and incubated for 24 h at 41.5°C. The results were confirmed by cultural cultivation on Brilliance agar *Salmonella* agar (BSA, Thermo Scientific, Thermo Fisher Scientific GmbH). Using the results of the triple approach, the MPN (colony-forming unit (**CFU**)/mL or g sample) was calculated using an MPN software program (available online: http://standards.iso.org/iso/ts/6579/-2/).

### Statistical Analysis

The Statistical Analysis System for Windows, the SAS Enterprise Guide, version 7.1 (SAS Institute Inc., Cary, NC) was used. For descriptive statistics, the means and standard deviation (**SD**) of *Salmonella* count for all 3 serovars inoculated in different matrices during the production cycle at different incubation times were calculated using PROC MEANS. For the statistical evaluation of significant differences, assuming normal distributed data, a Ryan-Einot-Gabriel-Welsch test (simple ANOVA) was performed for checking significant differences of the data. The independent variables were the different matrices and depend variables are the different timepoints of counting the *Salmonella* isolates. Differences with a significant level of *P* < 0.05 were considered significant.

## RESULTS AND DISCUSSION

**Figure 2:**
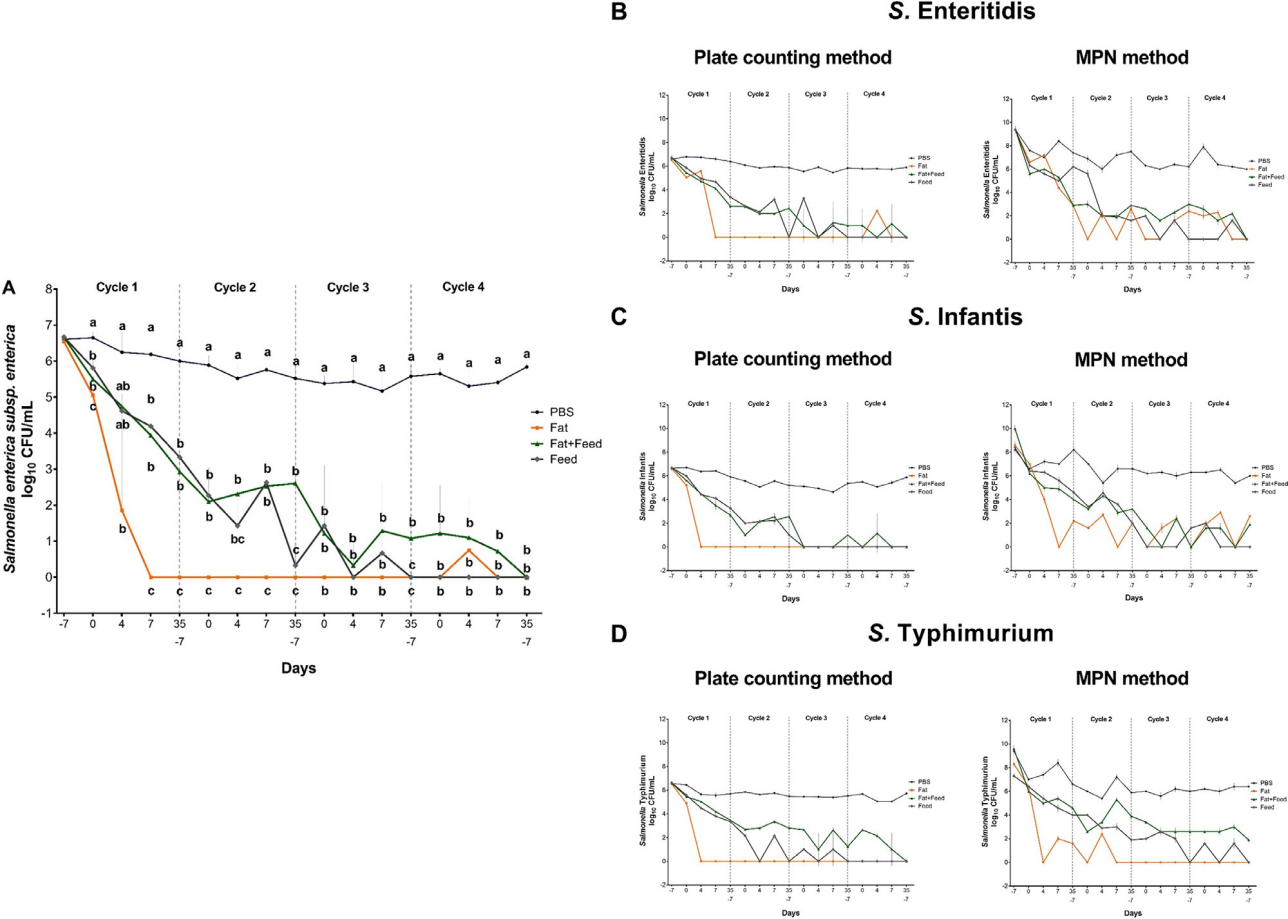
(A) Counts (log10 CFU/mL, means ± SD) of *Salmonella enterica* subsp. *enterica* isolates (all 3 serovars together, e.g., *S*. Enteritidis, *S*. Infantis, and *S*. Typhimurium) exposed to different samples (phosphate-buffered saline (PBS), fat, fat with feed, and feed) during the service period and production cycle at different incubation times, influence of sample, *n* = 3. Different letters (a, b, c) show significant differences between the matrices for each incubation time (P < 0.5). Abbreviation: SD, standard deviation. Survival of (B) *Salmonella enterica* subsp. *enterica* serovar Enteritidis, (C) *Salmonella enterica* subsp. *enterica* serovar Infantis, and (D) *Salmonella enterica* subsp. *enterica* serovar Typhimurium inoculated in different matrices (phosphate-buffered saline (PBS), fat, fat with feed, and feed) during the service period and production cycle/fattening period.

The current study confirmed the persistence of the 3 *Salmonella* isolates, *S*. Enteritidis, *S*. Infantis, and *S*. Typhimurium serovars for 4 consecutive production cycles (about 150 d). The result of *Salmonella* isolates together from all 3 serovars (*S*. Enteritidis, *S*. Infantis, *S*. Typhimurium) during the service period and production cycle at different incubation times are presented in Figure 2A. *Salmonella* showed the highest survival level in PBS matrices (*P* < 0.001), while the lowest *Salmonella* survival level was observed in fat matrices (*P* < 0.001). This is in agreement with Waldner et al. (2012) who reported that *S. enterica* can survive up to 30 mo in the environment without requiring an animal reservoir. In many studies, *Salmonella* strains isolated from poultry samples, for example, feces and litter samples show the ability to adapt to special conditions at different temperatures (Pulido-Landínez, 2019) and can survive in the environment without requiring an animal reservoir for long periods of time (Waldner et al., 2012).

In this study, all samples prior to the experiment were *Salmonella* negative. The artificial contamination with *Salmonella* trial proceeded as planned. In PBS, fat with feed, and feed matrices, qualitative detection was 100% *Salmonella* positive up until the end of cycle 4, while in fat matrices, negative results were found at d 7 of cycle 2 in all isolates. However, the results changed depending on the time points in each isolate. Interestingly, in *S.* Typhimurium isolate, negative qualitative detection was shown at d 7 of cycle 2 onward. The results of the quantitative *S*. Enteritidis, *S*. Infantis, and *S*. Typhimurium detection showed a reduction in *Salmonella* from the first day of artificial contamination to the end of the fourth cycle in all matrices. In agreement with our results, the temperature (20°C–35°C) at which matrices are kept has an effects the growth and persistence of *Salmonella*. Normally, *Salmonella* spp. are grown at 35°C to 37°C (Williams and Benson, 1978). It seems that the effect of temperature from d 0 to d 4 (d 0: 35°C and d 4: 31.5°C) in each fattening period might influence the growth and persistence of *Salmonella*. Williams and Benson (1978) mentioned that *S*. Typhimurium can survive for at least 18 mo when contaminated poultry feed is stored at 11°C, and the survival time thereof reached up to 16 mo when the storage temperature was increased to 25°C. Meanwhile, the survival time decreased to about 40 d when the feed storage temperature increased to 38°C. However, our findings changed depending on the time points in each isolate in each fattening period. The carryover of *Salmonella* serovars on broiler farms depends not only on farm management but also on the feed (Pulido-Landínez, 2019).

In accordance with the plate counting method (**PCM**), *S*. Enteritidis, *S*. Infantis, and *S*. Typhimurium were detected in PBS matrices with a high count in the first cycle (at d 0: 6.80 ± 0.11, 6.70 ± 0.11, 6.44 ± 0.07 log_10_ CFU/mL, respectively; Figure 2B-D) and did not greatly decline during all 4 cycles (at the end of cycle 4: 5.93 ± 0.00, 5.87 ± 0.02, 5.73 ± 0.05 log_10_ CFU/mL, respectively). Whereas in fat, fat with feed mixture and feed matrices, *Salmonella* could only survive for short periods of time. In the fat matrix, *Salmonella* declined to the lowest prevalence from d 4 onward for *S*. Infantis and *S*. Typhimurium and from d 7 for *S*. Enteritidis onward (0 log_10_ CFU/g; Figure 2B-D). While fat with feed as well as feed matrices, there was a gradual decrease in *Salmonella* count until the end of the second cycle. After that, fluctuations in the *Salmonella* count in the third and fourth cycles for all 3 serovars. At the end of cycle 4, in fat, fat with feed as well as in feed matrices, no growth of *Salmonella* for all 3 serovars were observed (0 log_10_ CFU/g). Previous studies revealed the ability of *Salmonella* when present in the feed to multiply in warm, moist surroundings, either at the feed mill or on the farm (Jones, 2011; Wason et al., 2021). Contrary to our findings, Wason et al. (2021) observed that fats tend to protect *Salmonella* from environmental or physiological stress, and in areas containing fat accumulation, *Salmonella* cannot be easily eliminated. Furthermore, Jones (2011) concluded that reducing oil or fat accumulation in the feeding lines is important to decrease the chances of *Salmonella* survival and spread.

Detecting *Salmonella* in feed can be challenging because low levels of the bacteria may not be recovered using traditional culturing techniques (Jarquin et al., 2009). Numerous detection methodologies have been examined over the years for quantifying *Salmonella* in animal feeds and some have proven to be more effective for *Salmonella* isolation and detection (EN ISO 6579:2002). Thus, in this study, 2 different methods, direct plate count on XLD agar and the MPN method were used to detect *Salmonella*. It is interesting that the MPN method revealed a similar reduction trend to that of the PCM (Figure 2A–C). Reductions in *Salmonella* from the first day of artificial infection until the end of cycle 4 were observed. However, the results fluctuated between the time points in each cycle. When comparing the *Salmonella* counts these were higher with the MPN method than the PCM estimations. Blodgett (2006) stated that the MPN method was more sensitive than the plate count method for detecting small numbers of *Salmonella*. In addition, MPN is useful for detecting low numbers of *Salmonella*. However, our results contrast with those of Madsen (1994) who reported that direct plate counts on XLD agar were consistently higher than MPN estimations. The higher effectiveness of the MPN method in detecting *Salmonella* could be demonstrated due to the use of pre-enriched triplicate samples from 10-fold serial dilution in addition to utilization of growth medium MSRV agar (Blodgett, 2006).

Additionally, as cross-contamination can occur at field level, feed weighers and the extractor hood are difficult to clean and disinfect and *Salmonella* are apparently not always eliminated by the cleaning and disinfection process (Zeng et al., 2021). If cleaning and disinfection are unable to eliminate *Salmonella*, the pathogen can remain and be transferred to the flock as well as newly hatched 1-day-old chicks via the feed and water lines (Zeng et al., 2021). Moreover, it would be interesting to simply examine the microflora of the feed lines/scales, since there are many factors that can affect the health status of young animals (Jones, 2011; Zeng et al., 2021).

The present investigations demonstrate that the persistence of *Salmonella enterica* subsp. *enterica* such as *S*. Enteritidis, *S*. Infantis, and *S*. Typhimurium in poultry feed-related matrices for a period up to 5 mo (4 production cycles) that may be encouraged by recontamination with *Salmonella* in broiler flocks. In addition, the results demonstrated that there were a fluctuation in growth of *Salmonella* which may be linked with change of temperature during each production cycle. Further research is needed to determine different survival abilities of *Salmonella* spp. in feeding pipes. Considering the survival ability of *Salmonella enterica* subsp. *enterica* serovars in feed residues during cleaning and disinfection processes of poultry houses is highly recommended.
